# Real-life impacts of olipudase alfa: experiences of adults receiving enzyme replacement therapy for acid sphingomyelinase deficiency—results from an international survey study

**DOI:** 10.1186/s13023-025-03997-6

**Published:** 2025-09-30

**Authors:** Adel Sabet Morsy, Solomon Mbua, Toni Mathieson, Justin Hopkin, Shaun Bolton

**Affiliations:** 1 International Niemann-Pick Disease Registry, Suite 2, Vermont House, Washington, NE37 2SQ UK; 2 Niemann-Pick UK, Suite 2, Vermont House, Washington, NE37 2SQ UK; 3 National Niemann-Pick Disease Foundation, Fort Atkinson, WI 53538 USA

**Keywords:** Acid sphingomyelinase deficiency, Olipudase alfa, Enzyme replacement therapy, Niemann pick diseases, Visceral symptoms, Neurological symptoms.

## Abstract

**Background:**

Acid sphingomyelinase deficiency (ASMD) is a rare lysosomal storage disorder caused by *SMPD1* mutations, resulting in sphingomyelin accumulation and diverse manifestations. Olipudase alfa, an enzyme replacement therapy, has shown efficacy in treating non-neurological symptoms of ASMD, while its impact on patient-reported outcomes remains underexplored. Therefore, there is a need to investigate the disease burden, patient perspectives, treatment expectations, risk tolerance, and unmet needs of adult ASMD patients receiving olipudase alfa.

**Methods:**

A retrospective case series design was employed, incorporating online surveys and semi-structured interviews. Surveys explored demographics, symptoms, and treatment experiences, drawing on input from stakeholders, including researchers, clinicians, and patient advocacy groups. Participants aged 18 or older with a confirmed ASMD diagnosis and receiving olipudase alfa were recruited through patient organisations. Surveys were administered online via Qualtrics, and interviews were conducted and transcribed for qualitative analysis.

**Results:**

ASMD posed substantial burden on participants’ ability to perform daily activities. Olipudase alfa was associated with substantial improvement in non-neurological manifestations of ASMD. Participants perceived the drug’s risks to be low, and the benefits outweigh the risks or burden. Most participants express satisfaction with olipudase alfa and their ability to lead better lives due to fewer ASMD symptoms since starting treatment.

**Conclusions:**

This study highlights the burden of ASMD and the positive impact of olipudase alfa on patients’ quality of life. Findings reinforce the importance of early diagnosis and accessible treatment. Despite the favourable outcomes, there remains a need for therapies targeting neurological manifestations and reducing treatment burden. Future research should focus on long-term outcomes and continue to prioritise patient-reported experiences to guide therapeutic development.

## Introduction

Acid sphingomyelinase deficiency (ASMD, Niemann-Pick Types A, B, and A/B) is a rare autosomal-recessive lysosomal storage disorder (LSDs) characterised by the accumulation of sphingomyelin and associated lipids within cells and tissues and presents with a broad phenotypic spectrum [1, 2]. The build-up accumulated lipids results from mutations in the *SMPD1* gene, which encodes acid sphingomyelinase (ASM), leading to reduced ASM activity and impaired sphingomyelin metabolism. ASMD encompasses a spectrum of clinical presentations which are often characterized into three general phenotypes:: acute neurovisceral (Niemann-Pick disease type A), chronic visceral (Niemann-Pick type B), and chronic neurovisceral (Niemann-Pick type A/B) [3]. Phenotypic manifestations vary, with type B typically lacking central nervous system involvement [4], while type A/B shows milder neurological symptoms than type A [5]. Type A is marked by severe neurodegeneration resulting in early death in the first few years of life. Symptoms of type A include irritability, sleep disturbances, failure to thrive, hypotonia, and severe gastrointestinal and respiratory issues [6, 7]. Type A/B patients may face premature death due to pulmonary infections or liver disease [6, 8]. In contrast, type B symptoms can appear from infancy to adulthood, with most patients exhibiting symptoms in early childhood. In adulthood, clinical features include hepatosplenomegaly, thrombocytopenia, pulmonary and cardiac disease, and skeletal issues. Premature mortality often results from cardiac, respiratory, or liver complications, or haemorrhage [7, 8].

Birth prevalence estimates of ASMD vary, with a 2015 study [9] involving six countries reported a live birth prevalence ranging from 0.25–0.6/100,000. A Chilean study of 1,700 healthy individuals found a heterozygote frequency of 1:105.7 for the common *SMPD1* variant p.Ala359Asp, implying a higher incidence of 1:44,960 [10]. Type A is more prevalent among the Ashkenazi Jewish population, while type B has a pan-ethnic distribution [10, 11].

Olipudase alfa, an enzyme replacement therapy (ERT), has recently been approved in several countries to treat the non-central nervous system manifestations of ASMD [12] and involves bi-weekly infusions of recombinant human ASM [13]. Clinical trials have shown positive outcomes in paediatric and adult ASMD patients, including reductions in spleen and liver volume and improved lung function and lipid profiles [14, 15].

Despite these promising results, further investigation on olipudase alfa’s impact on patient-reported outcomes is needed. To date, the adult experience with this therapy has not been comprehensively studied. This study, conducted by the National Niemann-Pick Disease Foundation (NNPDF), Niemann-Pick UK (NPUK), and the International Niemann-Pick Disease Registry (INPDR), aims to explore: (1) ASMD disease burden, (2) patient and caregiver perspectives, (3) what makes a therapy meaningful, (4) patient risk tolerance levels, and (5) unmet therapeutic needs of ASMD patients.

## Method

A retrospective case series study was conducted, utilising online surveys and semi-structured interviews to procure qualitative and quantitative data. The surveys were co-designed by researchers, clinicians, patient advocates and patient families, and considered the known natural history of the disease [7, 10]. Data collection and surveys were conducted by Rare Disease Research Partners (RDRP) on behalf of NNPDF, NPUK and INPDR.

### Recruitment

The study was advertised via email and social media by NNPDF and NPUK amongst their members and various other national member organisations. The survey was open to all potential participants, whilst the interview was open to participants who have previously participated and completed in the survey (Table 1). Survey participants who wished to take part in an interview provided their consent to be contacted by the RDRP, who arranged the interview session.

**Table 1:** Participant inclusion criteria for (a) survey and (b) interview

***(a) Survey inclusion criteria***
Aged 18 years or over
Fluent in English (including non-native English speakers)
Able to give informed consent
Confirmed diagnosis of ASMD
Currently receiving treatment with olipudase alfa or previously received treatment with olipudase alfa within 6 months of study participation
Consented to participate in the study
***(b) Interview inclusion criteria***
Has participated and completed the survey
Consented to participate in the interview

This research was conducted in accordance with the British Healthcare Business Intelligence Association’s Legal & Ethical Guidelines for Market Research. Monetary incentives were not provided to participants.

### Study design

The online survey aimed to explore the ASMD patient experience, encompassing demographics, initial symptoms, pre- and post-treatment symptoms, overall symptom progression post-treatment, and experiences with olipudase alfa. Considering the absence of validated tools for assessing patient-reported outcomes (PROs) in ASMD patients and their families, key stakeholders including opinion leaders, researchers, patients, families, and advocacy organisations were consulted to design survey questions. The survey included a mix of multiple-choice, matrix, and open text questions to provide qualitative and quantitative data.

Certain survey queries closely resembled the Splenomegaly Related Score (SRS) from the adult ASCEND trial, aiming to evaluate disease burden potentially associated with organomegaly [15].

The survey was administered through the QualtricsXM [16] online platform and shared via a link. It remained accessible for responses from February 7th to May 12th, 2023.

A semi-structured interview guide was crafted to delve deeper into the survey inquiries and gain comprehensive insights into the effects of olipudase alfa on patients and their families. Interviews were conducted via the Zoom platform by Rare Disease Research Partners. The sessions were recorded in audio format and subsequently transcribed for analysis.

### Data analysis

Qualitative and quantitative methodologies were employed to analyse the survey data. The transcripts from the interviews were scrutinised using an inductive thematic approach facilitated by NVivo software.

## Results

### Quantitative results

#### Participant characteristics

Twenty-one participants attempted the survey between 07 February–12 May 2023, of which 11 responses were included in statistical analysis. Of the excluded participants, seven did not complete the full survey, two participants were found to be duplicates and one did not meet the eligibility criteria (< 18 years).

Except one caregiver, all participants were ASMD patients residing in the United States, Canada, Spain, United Kingdom, and South Africa. US and Canada based participants took part in interviews. The median age of the 11 ASMD participants at the time of the survey was 42 years (mean 9.8, range 20–57). 73% of participants were female. All participants were diagnosed with ASMD after birth, and 82% of participants were diagnosed after symptoms of ASMD appeared. Diagnosis was made by enzyme testing (*n* = 1), DNA sequencing (*n* = 1), both enzyme and DNA sequencing (*n* = 2), or tissue biopsy (*n* = 4) at a median age at of 7.5 years (range 2–36). Three participants did not report their method of diagnosis.

#### Burden of ASMD symptoms prior to treatment and during treatment

Age at first symptom onset was reported between two months and two years of age for 46% of participants, with 82% reporting first symptoms before 18 years. 45% reported neurological symptoms including neuropathy (18%), ataxia, impaired cognition or learning disability, memory loss and multiple sclerosis (all 9% each).

**Fig. 1:**
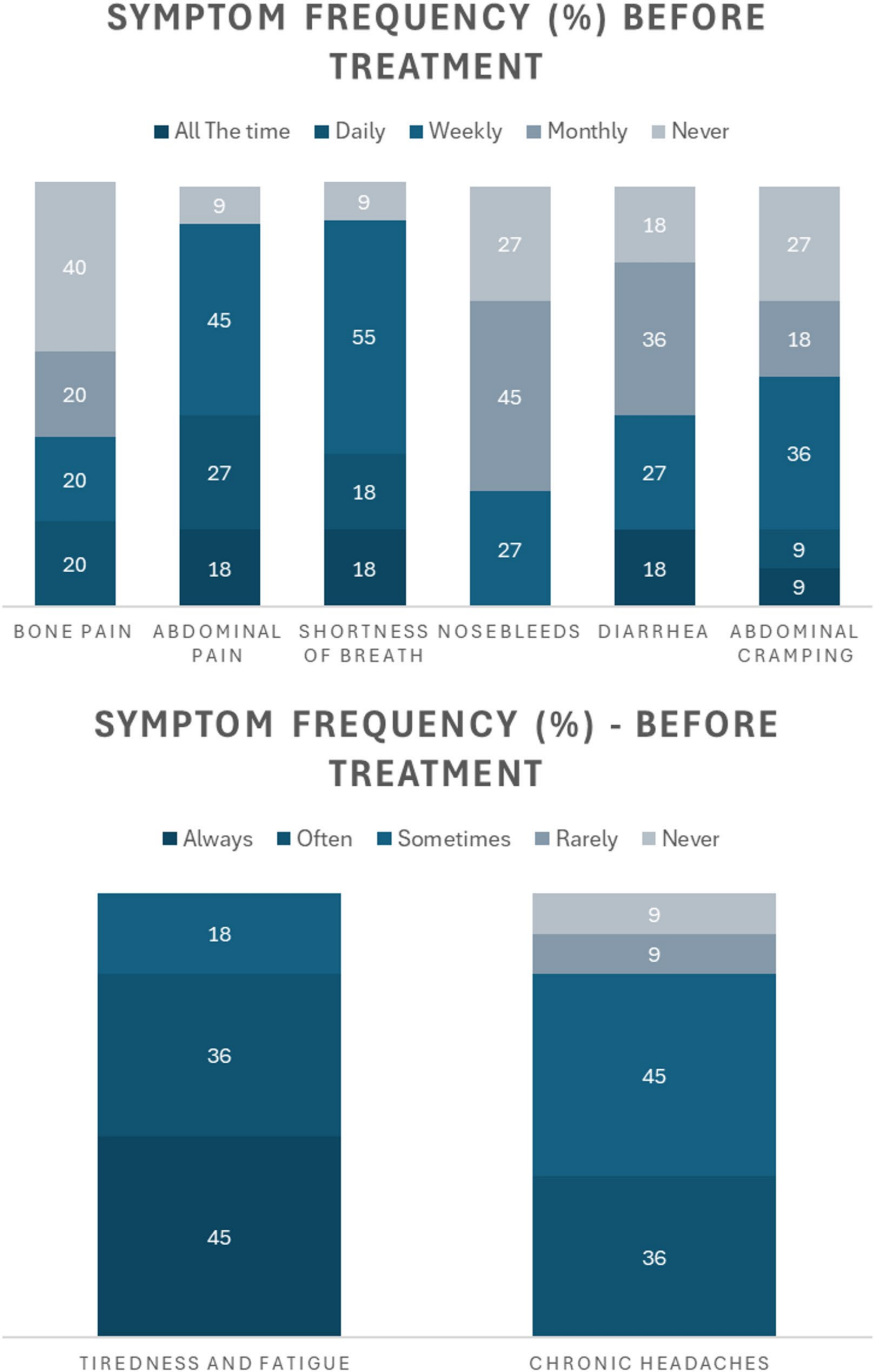
Reported symptom frequency experienced by participants prior to treatment with olipudase alfa

When asked about six commonly occurring ASMD symptoms, most participants experienced each symptom with varying frequency (Fig. 1). The majority of participants experienced abdominal pain and shortness of breath, with 18% reporting experiencing both symptoms ‘all the time’. Furthermore, all participants reported regularly experiencing tiredness and fatigue at differing frequencies.

**Fig. 2:**
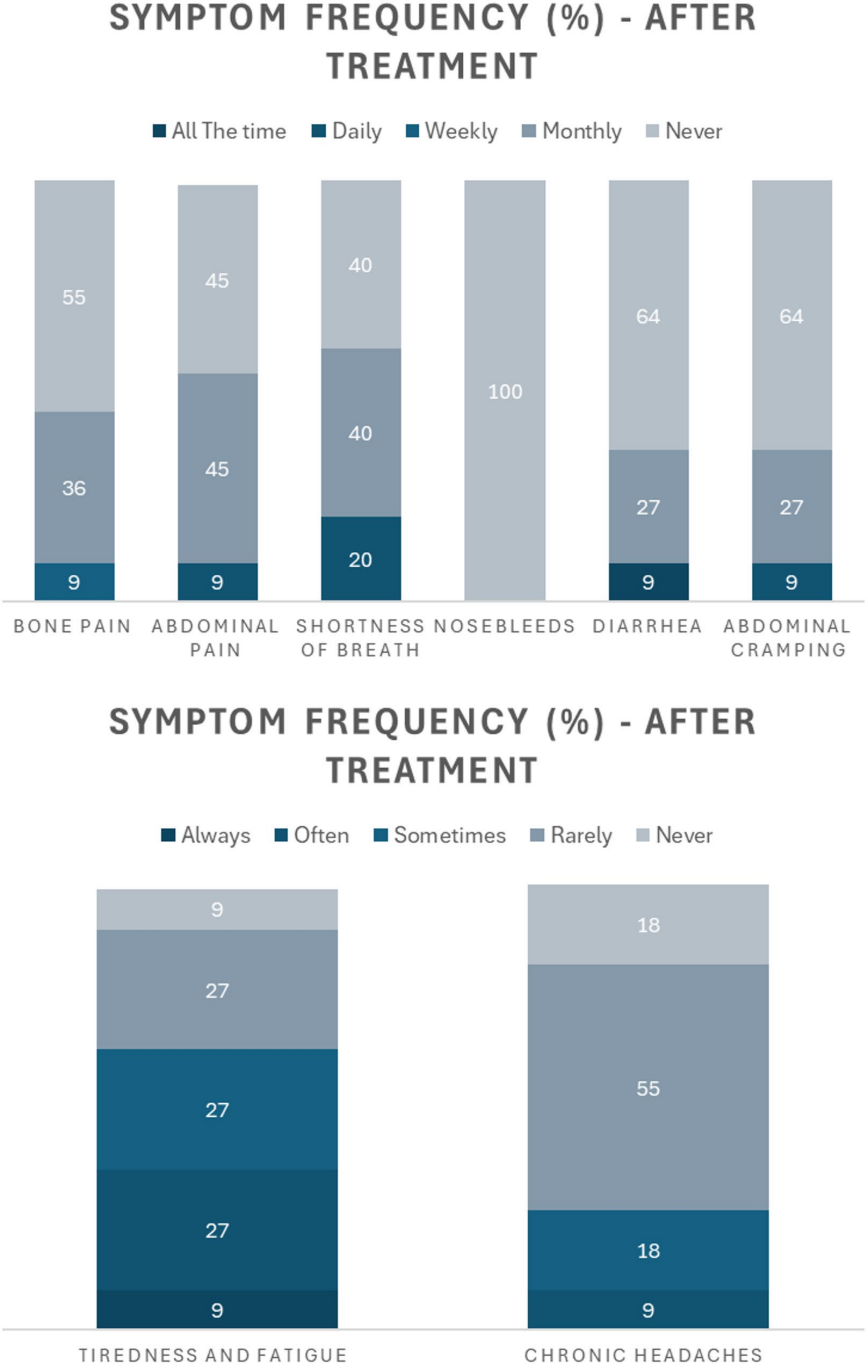
Reported symptom frequency experienced by participants after treatment with olipudase alfa

After treatment with olipudase alfa, participants reported frequency reductions across each ASMD symptom to varying degrees (Fig. 2).

#### Impact on daily life

Participants were asked about the impact of ASMD on their daily lives before starting treatment with olipudase alfa. Many reported taking time away from school/work due to medical appointments (73%) or ASMD symptoms (55%). 27% reported an inability to take part in extracurricular activities, with 27% also reporting school/work was awkward because of ASMD.

In terms of physical activity and sports involvement, a substantial portion of participants expressed challenges with 55% indicating they were unable to participate at all, 27% reporting some limitations, and 18% reporting only insignificant barriers.

Mental health concerns due to ASMD were prevalent before treatment, with 73% expressing worry and anxiety and 45% feeling frustration. Additionally, 36% reported feelings of isolation, 27% noted various social and emotional impacts, while 18% reported no specific challenges.

Regarding thoughts about future apprehensions related to ASMD, many participants expressed a general uncertainty about what lies ahead (73%), while 64% harboured fears of losing physical abilities, and 27% worried about increasing social isolation from loved ones. Furthermore, 18% expressed concerns such as difficulty finding a life partner due to ASMD, and fears of passing the condition on to future generations, while 18% reported having no specific worries.

**Fig. 3:**
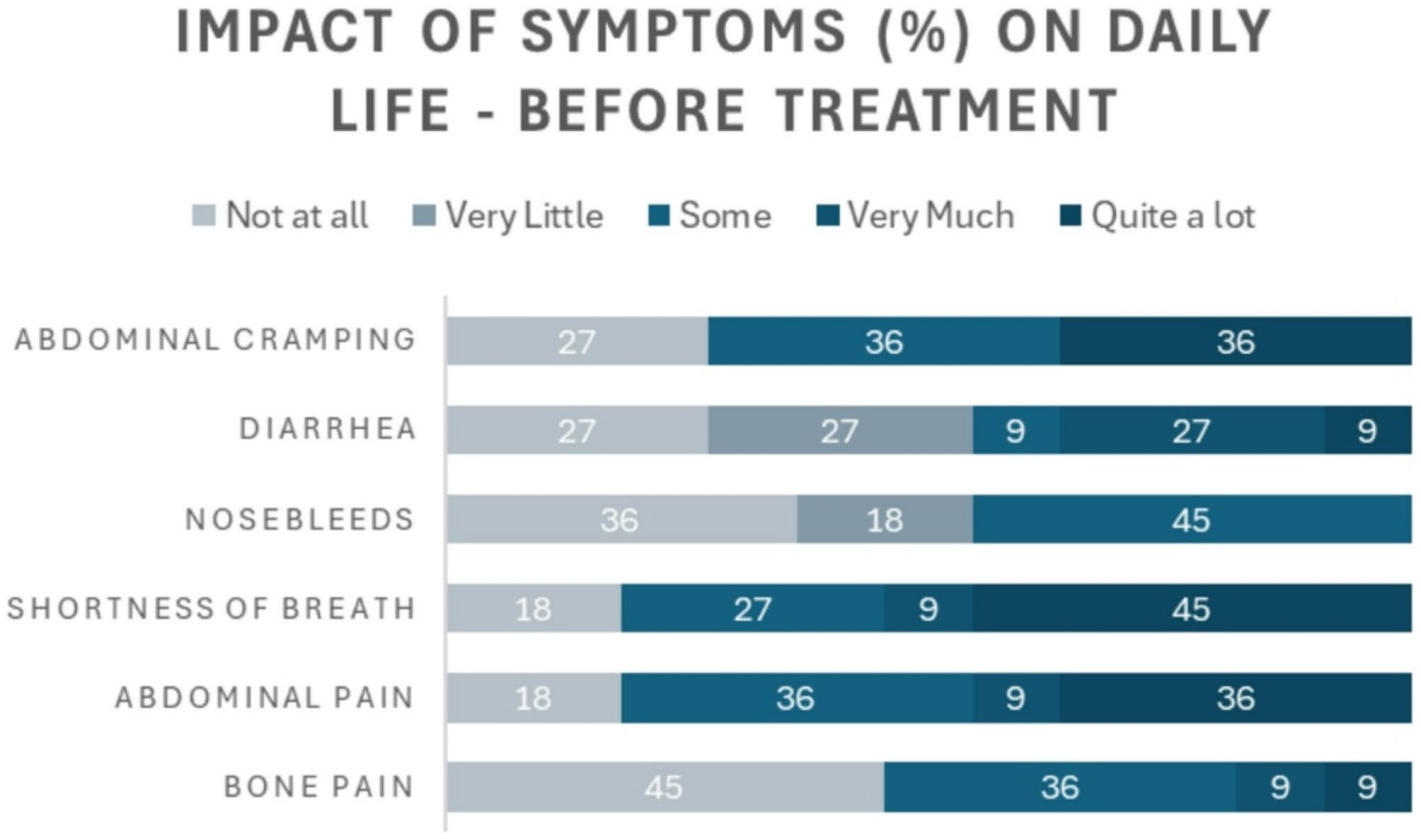
Impact of symptoms on day-to-day life of participants before treatment with olipudase alfa

**Fig. 4:**
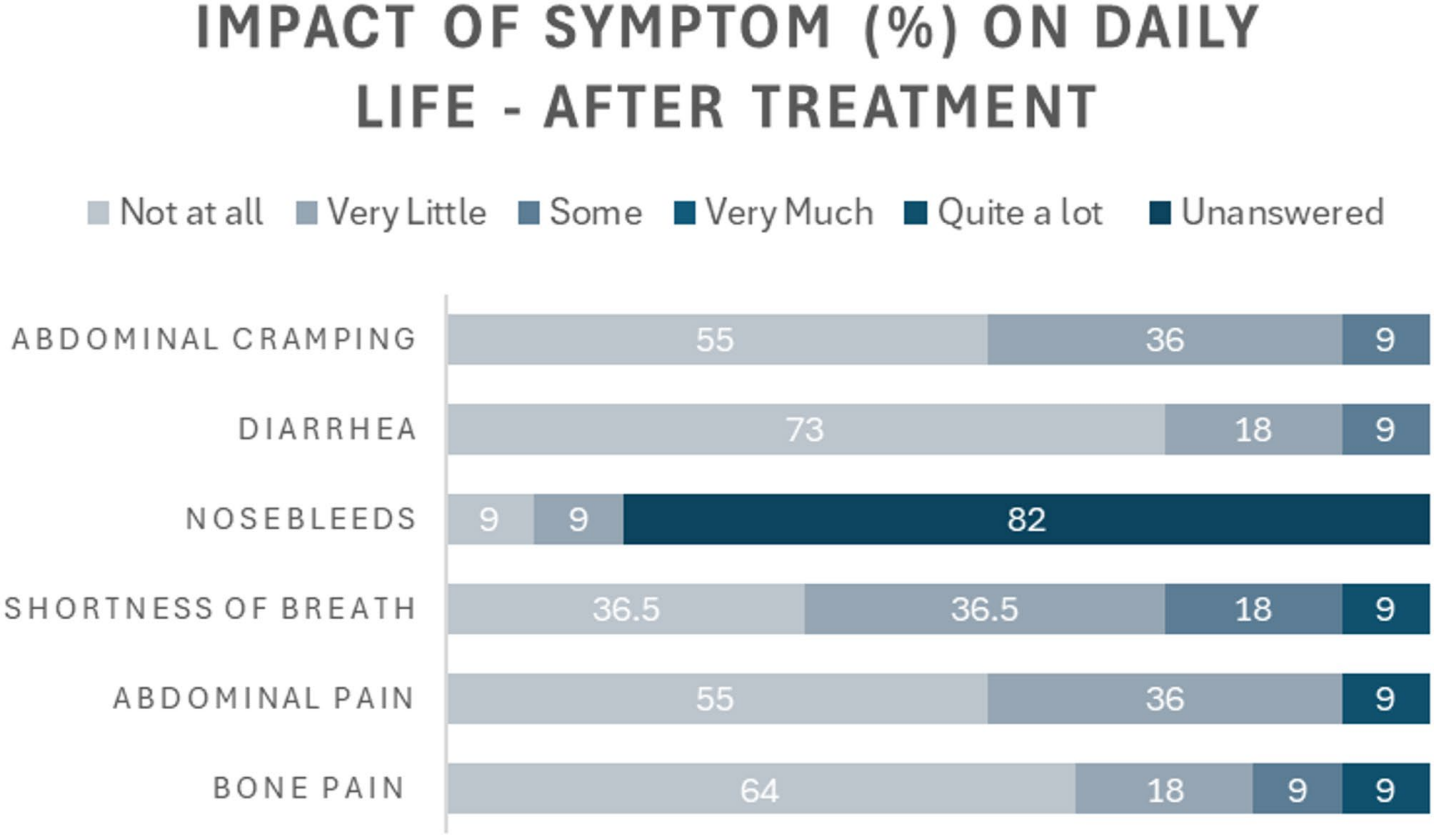
Impact of symptoms on day-to-day life of participants after treatment with olipudase alfa

In respect to ASMD symptom impact prior to treatment, shortness of breath, abdominal pain, and abdominal cramping were reported to be most impactful symptoms (Fig. 3). Meaningful reductions across each symptom’s impact on daily life were reported after initiation of olipudase alfa (Fig. 4).

#### Global impression of change

**Fig. 5:**
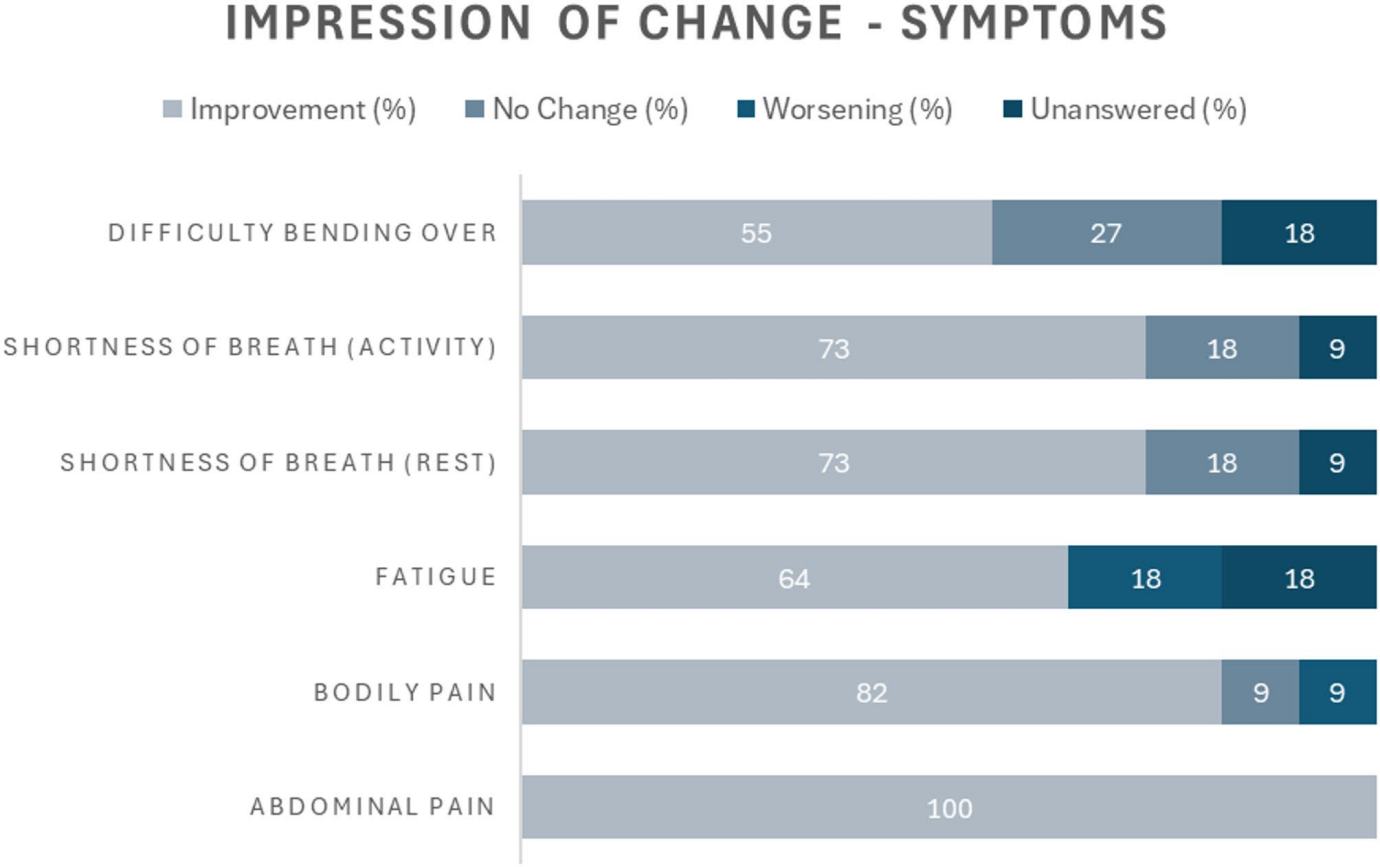
Participants’ impressions of changes in ASMD symptoms following treatment with olipudase alfa

**Fig. 6:**
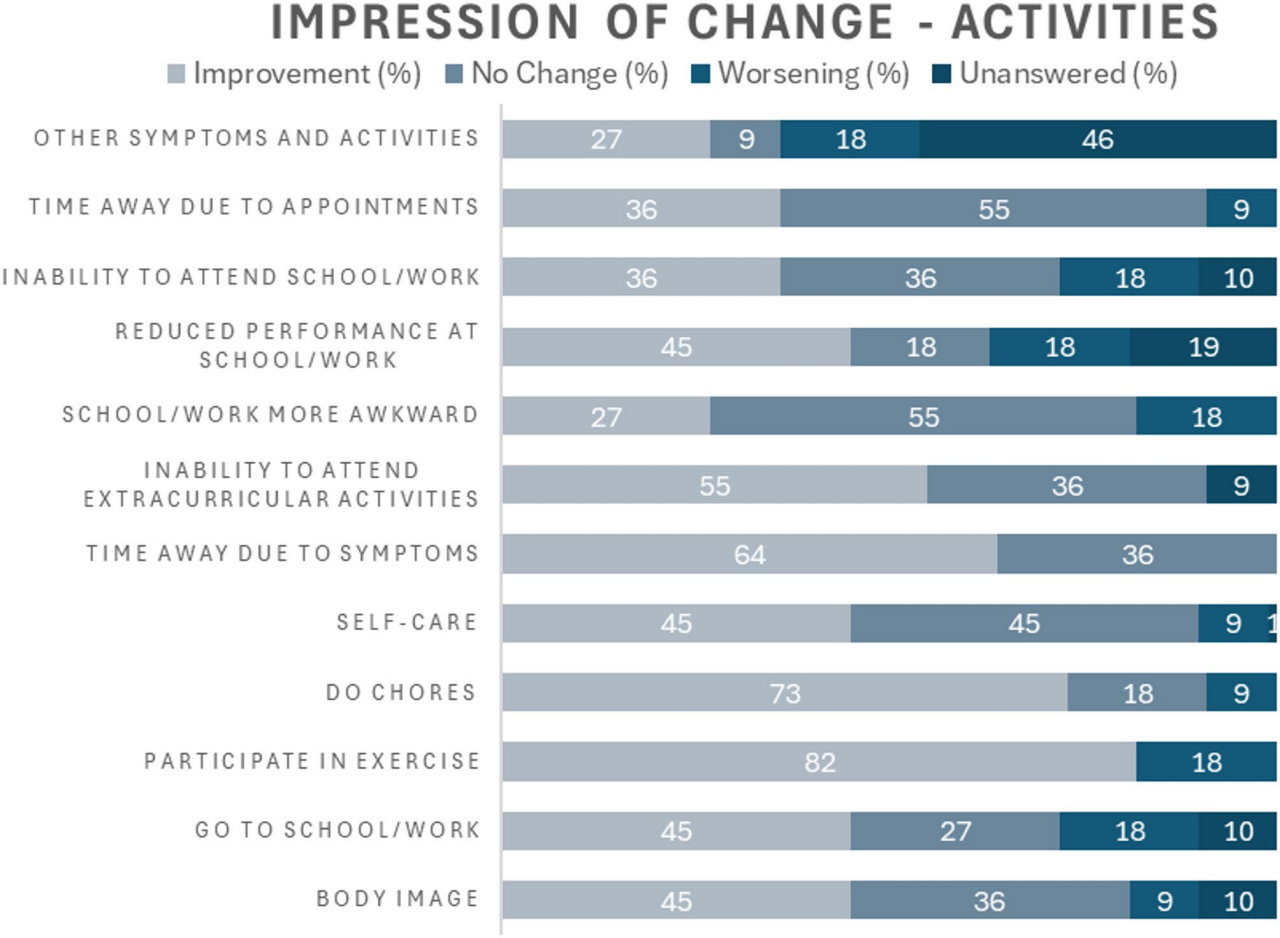
Participants’ impressions of changes in activities following treatment with olipudase alfa

Since commencing treatment with olipudase alfa, improvements across all symptoms and activities were reported, with all participants reporting improvement in abdominal pain (Fig. 5). Regarding activities, most participants reported improvement in participation in exercise and doing chores (Fig. 6).

#### Satisfaction of care

Participants were questioned about their satisfaction with olipudase alfa. 64% were extremely satisfied with its management of ASMD symptoms, whilst 27% were somewhat satisfied and 9% were somewhat dissatisfied.

### Qualitative results

The data of five participants interviewed as part of this study was analysed, and their experience reflects a large baseline disease burden. Both participants and families reported olipudase alfa had a positive impact on burden of disease.

#### Experience of ASMD in childhood and adolescence

Participants reported a negative impact of ASMD in various aspects of their childhood and adolescence. Below are some of the areas that were highlighted including several reports of how the disease impacted the entire family.

Impact on ability to attend school or work: *“And at the time*,* they wanted to put me into a special needs school for children who had more learning disabilities and things like that. Because the primary school didn’t want to take responsibility for the fact that my abdomen was fragile.*

*And also*,* there was this element of risk*,* if I was in normal school*,* getting pushed about*,* knocked over. So*,* my parents had to fight hard to get me into mainstream school. And then when I was in mainstream school*,* I wasn’t allowed to do anything that was remotely physical with any of the other children. So*,* playtimes I was segregated. I wasn’t allowed to play with them. I wasn’t allowed to do sports. I wasn’t allowed to do any form of PE or Sports Days. They used to find me other things to do like puzzles and handwriting club.”*

Impact on mental health: *“Certainly*,* growing up*,* the enlarged liver and spleen*,* which meant that I couldn’t be part of physical education classes and looked different than everyone else. And getting clothes to fit was a challenge. The first time I ever got asked if I was pregnant*,* I think I was only eight or nine*,* by a clown. And I’ve developed a significant aversion to clowns.”*

[…] and then you get where kids also don’t understand, and you get bullied because you look like you’re pregnant when you’re a little kid. I was, not now, but I used to be, real tiny, tiny, like you could look at me, I looked like a little elf, but I had a large belly.

Impact on families: *“So*,* from my family’s point of view*,* it put a lot of stress on our relationship as a family. Because at that point there was no cure*,* there was no treatment*,* and we didn’t know anybody who lived beyond 40*,* 45. Everybody I know who I grew up with were either really poorly with it or had passed away.”*

I know that my mum was an excellent advocate, who went to bat for me on many occasions. My dad, unfortunately, was an alcoholic, and I do not know whether the stress of this contributed to that. He stopped drinking completely when I was in university, but I suspect the stresses added to that, in terms of that as being a coping strategy for him at that point.

#### Symptom progression

Participants reported several progressive ASMD-related symptoms with shortness of breath, fatigue, and enlargement of the liver and spleen being the most reported signs and symptoms.

Spleen and liver: *“Yes*,* so I’m badly affected in comparison to the other people that I know. I developed a lot of storage*,* and so from the age of diagnosis through to about 12 years old*,* my condition progressed quickly. My abdomen got big*,* distended*,* my spleen was from not long after diagnosis*,* around three*,* four years old was markedly distended and wasn’t functioning properly. I was bruising all the time as a toddler*,* my stomach was constantly upset because of the thickening of my gut. So*,* I was constantly having upset stomachs all the way through primary school*,* well into secondary school.”*

Breathlessness and fatigue: *“Fatigue and shortness of breath have always been part of my picture with ASMD*,* but it had become progressive over time. Prior to starting on treatment*,* it had gotten to the point that in order to have enough energy to get through the work week*,* I could really cut out pretty much everything else in terms of social activities or things. I got the bare essentials done at home. I spent the weekends at home recharging in order to be able to get back through the work week*,* the following week.”*

#### Experience of ASMD in adulthood

Participants reported a significant impact of ASMD in various aspects of life in their adult years. ASMD-related signs and symptoms worsened with age, and new symptoms and challenges appeared. Three common themes emerged regarding the impact of the disease which are captured below.

**Impact on activities of daily living** *“When I run*,* it was real hard for me to breathe. I get shortness of breath. Just walking or doing a flight of stairs*,* I would be winded*,* or even*,* at times*,* sitting*,* having a conversation*,* having to talk long*,* long sentences. I would get winded even doing that.”*

**Impact on mental health** “It’s hard to be happy when you’re hurting.”

*“But*,* somehow*,* in my brain*,* I assumed that would go away as I got older. But of course*,* it didn’t. The tone of it changed as I got older because it became an age that it was socially acceptable if you’re pregnant*,* and people would say things that they thought were very positive*,* like oh*,* you look like you’re having a boy. And they were trying to do it in a very positive framework*,* but it just was like*,* really? Because I work in a hospital*,* I would have patients saying oh*,* I’m sure that’s a boy*,* and my colleagues are standing there going*,* no.*

**Impact on family and friends** *“I’d love to be there*,* an anniversary party for friends. I would absolutely love to be there*,* but it’s a two-hour drive one direction and a two-hour drive back*,* and I simply can’t. I don’t have the energy reserves to do it.”*

It really meant that I was using my energy and directing my energy and time to doing the absolute essentials. So, get the groceries, get the laundry done, get some meals, get through the workweek. I gradually cut out going out with friends.

#### Change in ASMD symptoms

One of the key themes identified in the interviews was an overall improvement in ASMD-related symptoms following treatment. Participants reported first seeing improvement in symptoms three weeks to one year after beginning infusions of olipudase alfa. These improvements include:

Infections: *“I have like an allergy sinus. Everyone’s dealing with it right now in [Location]*,* a sinus infection type of thing. I haven’t been sick in seven months*,* thank God. I’m dealing with it right now. Seven months is good not being sick.”*

Appetite: *“[…] I was eating food and gaining weight. And not only that*,* I didn’t need to eat a lot. I was eating a normal meal*,* and then I was fine for four*,* five*,* six hours. I wasn’t even hungry.*

*Whereas before*,* I would have eaten that and then 20 min later looking for something else to have to eat. So*,* my meal sizes had cut down.”*

There were participants who also reported that their ASMD-related symptoms had slightly improved or had not progressed further. These symptoms include:

Headaches: *“So*,* I take ibuprofen*,* but I don’t get them as much as I used to*,* but I still get them once in a while.”*

Nosebleeds: *“But now*,* since the treatment*,* I might get a nosebleed maybe every six months and it would just be the one*,* and that’s it. […]. It’s better now*,* yes. I mean*,* I had a nosebleed maybe about two or three weeks ago*,* I had one. Or did I have two? I think I had one. But it was the first time in about six months I’d had one.”*

#### Overall change in quality of life

One of the most important impacts of treatment to participants was a positive influence on their health and quality of life. Participants reported feeling their health was better and could see a difference in themselves from prior treatment.

It’s overall beneficial that I’ve taken it. My health has overall improved. It’s beneficial. Everything has gotten better, overall, since I’ve been on it for ten years. I can see with my bloodwork, my MRIs, my CAT scans, everything in general. Even doing things, like I’m more active. I can see a difference in myself, overall, from prior to taking olipudase alfa.

I feel better in general. I still have my good days and my bad days. That’s the only thing. I was hoping that I’d feel good all the time. I’ll take the good and the bad.

It’s a massive positive. […]. And when I started the enzyme replacement therapy, my health flipped, completely turned on its head. All these things I’ve never been able to do I could do. […]. Because the only thing that’s giving me my quality of life right now is the enzyme replacement therapy.

In terms of the impact on day-to-day life, before treatment, some participants had left education, left employment or were considering leaving employment due to their ASMD. For those who were still in education and employment, after treatment began, they were able to cope better with work and continue their education.

I think it improved my ability to be able to continue at work. I think it was getting to a point where I was deciding whether I could or couldn’t continue in this. Several of my colleagues had asked about would I consider looking at a non-clinical role because of the demands of my clinical role. So, I certainly think it has started to make a positive impact on my ability to maintain that.

Participants were less vulnerable to infections, which meant less days off work due to illness and one participant detailed how they were able to continue working throughout the COVID pandemic after being removed from the shielding register because of their good health.

*“The guy I work with has had coronavirus three times*,* brought it into work and then gone home on the sick. And I’ve never caught it. And although we’re using a lot of protection*,* years ago that probably would have killed me*,* to be perfectly honest. I certainly wouldn’t have been working.*

*And my consultant took me off the shielding register because I was immediately shielded when the coronavirus started. And within about six months of the initial start*,* he had me removed from the shielding register because my health was as good as anybody else. And I returned to work and worked all the way through it.”*

Since beginning treatment, participant’s mental health had improved as their health had improved and they felt less negative about living with ASMD.

Yes. I think, again, a biproduct of just having the treatment is that it affects my mental health in that I know I’m being treated and that, in itself, improves my mental health, because I know my physical health is being improved.

I’m happier that taking the enzyme treatment, overall, I’m very happy that I decided ten years ago to take this enzyme treatment.

#### Disadvantages of treatment with olipudase Alfa

Disadvantages of treatment were mainly related to infusions and their planning. Impact on daily life was most acute during the clinical trial, after which treatment became more manageable, although life often had to be planned around them. One participant mentioned the impact on planning a family.

**Infusions** *“I don’t really have concerns about the actual drug*,* it’s more the IV process. Because I don’t know if you can see*,* I’ve got a big bruise. That’s it.”*

Yes, I do take the day off work on that day every fortnight, purely because it’s… Maybe I could work whilst having the treatment, but pretty much one of my arms is out of commission and it does make me very lethargic, the treatment. Very unmotivated.

**Participating in the clinical trial** *“During the clinical trial I had to plan around*,* because in the clinical trial they had a big thing about you can’t have alcohol the day before*,* the day of*,* three days after. So*,* if it’s going to be New Years*,* or your birthday*,* or somebody’s wedding*,* and you might want to have a drink. It would be like*,* ‘I have to move my infusion’. And at first*,* we couldn’t move our infusions around.”*

**Planning a family** *“I can’t have kids*,* so that’s it. That was the only other thing. That’s the only worry. I asked many times throughout the research and they never really gave me an answer about children and stuff like that. I think it’s passed that. It’s the only other thing. They actually came out and said that you’d have to stop to have children. I wish they had told me that before. I would have planned a little bit differently before I would have started. I had a couple of months*,* maybe I would have planned it a bit differently.”*

#### Participants’ outlook on the future

Participants’ perspectives on their future health shifted because of their treatment. Participants believed that if treatment stopped, their future health could be compromised. Due to the perceived benefits, they believed in a need for early and accessible treatment.

**Outlook on future health** *“As long as I can keep receiving the treatment*,* I feel more optimistic about my future when it comes to my health than before I started the treatment. I was told or made aware before I started the treatment that my health would decline as I got older. And that’s less of a factor now that I’m being treated. That’s what I would say.”*

With regards to my health, now I know that I do have a future. Before, I was actually worried about whether I would have a future. I lost another friend at 27 from a spleen aneurysm, so I didn’t know if anything like that was going to happen to me. Like the splenic infarct, I didn’t know if it would come back.

**Impact if treatment were to be stopped** *“I would get sick. I wouldn’t feel good. It would be devastating.”*

Yes. And if it was taken away from everybody, I’d be depressed about the other people. I had a couple of friends that died before it got approved.

**Access to treatment** *“Yes. I think it is something that everyone should have made available to them if they have ASMD. There are people who are affected with ASMD worse than I am who would benefit greatly from the treatment. I know several others who are on the treatment as well who maybe benefitted more than I do because of the severity of their symptoms. So yes*,* I think it should be made… If they can receive the treatment*,* they should.”*

**Need for early treatment intervention** *“Oh*,* I think it’s extremely important because I watched*,* being little and being diagnosed with it and living with it my whole life and how it progressed over time. I think it’s extremely important to have that because if you can catch it early on*,* before anything starts getting crazy and get on the medication*,* you can have*,* I guess*,* more of a*,* what people say*,* normal life. But you won’t have to experience all the crazy stuff either*,* as far as you could play those contact sports.”*

**Need for treatment alternatives** *“Another treatment? So*,* I feel like there is*,* only because [Name] said olipudase alfa will not pass through to my brain. But they’re working on gene therapy*,* taking my bad gene and giving me a good gene*,* so yes.”*

*“Okay. Well*,* just thinking of what I would’ve been thinking then*,* it is still being attached to a drip for four hours. If it was a pill I took every day*,* then I would be completely satisfied.*

## Discussion

This study investigated the effects of olipudase alfa on the disease burden for adult ASMD patients or their caregivers, focusing on perceived benefits, risks, and unmet therapeutic needs.

### Summary of findings

The study’s quantitative and qualitative results suggest olipudase alfa positively impacts both psychological and physical health outcomes, aligning with findings from the associated paediatric study [17]. Consistent with prior literature, the surveyed group reported significant lifelong ASMD symptoms, with nearly half experiencing symptoms as early as 2 months to 2 years old [7, 18]. These findings highlight the critical importance of early diagnosis and timely intervention [19].

### Impact of disease

Untreated ASMD imposes a continuous burden on adults, with many missing school or work due to symptoms and medical appointments, highlighting the disease’s disruptive nature. The inability to participate in extracurricular activities, along with symptoms like shortness of breath, abdominal pain, and cramping, restricts physical and social engagement, ultimately lowering quality of life [16, 18]. Prior studies [18, 19] show that these restrictions exacerbate mental health issues, with worry and anxiety further illustrating ASMD’s psychosocial impact.

Qualitative analysis revealed that ASMD symptoms profoundly affect daily activities, social interactions, and quality of life. For instance, two participants reported social withdrawal due to self-consciousness over their protruding abdomens, leading to bullying and avoidance of sports due to injury fears. Fatigue and vulnerability strained relationships, impacting parental mental health, while siblings often took on caregiving roles, sacrificing their own childhoods and missing milestone events [18, 20]. Further research is needed to understand rare diseases’ full impact on family wellbeing.

### Future health

Participants voiced concerns about their future health, fearing physical decline and social isolation—an apprehension supported by a recent systematic review assessing the psychological effects of LSDs [20]. This reinforces the need for comprehensive care strategies that address both current symptoms and long-term health prospects, as supported by various studies [7, 18, 20].

### Impact of olipudase Alfa

Overwhelmingly positive improvements in symptoms and daily activities following initiation of olipudase alfa treatment were reported. The reduction in abdominal pain and other symptoms suggest that olipudase alfa is effective in mitigating some of the most burdensome aspects of ASMD. This positive change in daily life and symptom frequency is consistent with previous literature [15, 17] and indicates not only symptomatic relief but also an enhanced quality of life for patients. Qualitative results also show that patients’ perspectives on their future health shifted positively due to their treatment. Participants believed that discontinuing treatment could lead to a decline in their health and stressed the importance of access as well as early intervention to prevent the progression of ASMD to enable a more normal life, including participation in activities like contact sports, which has also been documented in a 2009 ASMD type B study [19].

### Satisfaction of care

While satisfaction with olipudase alfa was high, logistical challenges from regular infusions prompted some participants to express interest in more convenient treatment options, such as oral formulations or gene therapy. Only 9% of participants indicated dissatisfaction, suggesting opportunities to further optimise treatment protocols such as daily pills.

### Clinical and research implications and future studies

Rare disease trials often involve small participant numbers, making retrospective PROs crucial for understanding ASMD and its treatments. Combining qualitative and quantitative data provided insights into known morbidities and revealed additional symptoms, offering a holistic view of how interventions affect patients.

The findings highlight the importance of early diagnosis and timely intervention in ASMD, as well as improvements in quality of life with olipudase alfa, underscoring the need for accessible treatment. Additionally, this study amplifies the patient voice, demonstrating the physical and psychological toll of ASMD on quality of life. It contributes valuable knowledge for the international ASMD community to support discussions around care management and access to treatment.

Future research should pursue multiple priorities identified through this study. Firstly, monitoring of the long-term impact of treatment on patient-reported health outcomes and quality of life should be undertaken to identify potential long-term benefits and risks. Secondly, initiatives to reduce time to diagnosis, and by extension enable earlier disease specific clinical management, including initiating treatment where appropriate, should be developed. Thirdly, additional treatment modalities, including targeting of the neuropathic elements of ASMD, and reducing the lifestyle impact of treatment, should be developed.

### Strengths and limitations

The study’s strengths include its comprehensive data collection methods, combining quantitative and qualitative approaches, multi-stakeholder survey design, international recruitment, and absence of industry involvement in study design, execution and analysis. The extended time since treatment commencement (compared to the original trial [15]) at the time of the survey allows for a long-term assessment of the impact of therapy.

Limitations include a small sample size, small geographical distribution of participants, lack of disease subtype information, and potential selection bias from recruiting through patient advocacy organisations. To mitigate bias, an independent third party conducted and assessed interviews. Recall bias may have arisen from participants reporting symptoms prior to treatment. Additionally, common symptoms like fatigue and shortness of breath are not specific to ASMD, complicating attribution to the disease. Furthermore, the study was limited to English-speaking participants.

## Conclusion

This study provides valuable insights into the burden of ASMD and the positive impact of olipudase alfa on patients’ lives. It highlights the ongoing challenges faced by patients and caregivers and the critical need for accessible treatments and early intervention strategies. Future research should continue to explore patient-reported outcomes and develop therapies that address the full spectrum of ASMD symptoms, including neurological manifestations, and reduce treatment burden.

## Data Availability

The datasets generated and/or analysed during the current study are not publicly available due to maintaining participant confidentiality and anonymity but are available from the corresponding author on reasonable request.

## Abbreviations

ASMD: Acid sphingomyelinase deficiency
ASM: Acid sphingomyelinase
ERT: Enzyme replacement therapy
LSD: Lysosomal Storage Disorders
INPDR: International Niemann–Pick Disease Registry
NNPDF: National Niemann–Pick Disease Foundation
NPUK: Niemann–Pick UK
PE: Physical education
PRO: Patient–reported outcome
RDRP: Rare Disease Research Partners
SRS: Splenomegaly Related Score
